# Synthesis and Biological Evaluation of Metamorphine: A Morphine–Metamizole Adduct from Patient-Controlled Analgesia Pumps

**DOI:** 10.1021/acsptsci.4c00546

**Published:** 2025-03-03

**Authors:** Aly Abotaleb, Aurélien F. A. Moumbock, Rainer Trittler, Gernot Zissel, Stefan Günther, Martin J. Hug

**Affiliations:** 1 Pharmacy, Medical Center - University of Freiburg, Freiburg D-79106, Germany; 2 Institute of Pharmaceutical Sciences, Faculty of Chemistry and Pharmacy, University of Freiburg, Freiburg D-79104, Germany; 3 Department of Pneumology, University Medical Center and Faculty of Medicine, University of Freiburg, Freiburg D-79106, Germany

**Keywords:** metamorphine, morphine, metamizole, patient-controlled analgesia pumps, drug interaction, pain management

## Abstract

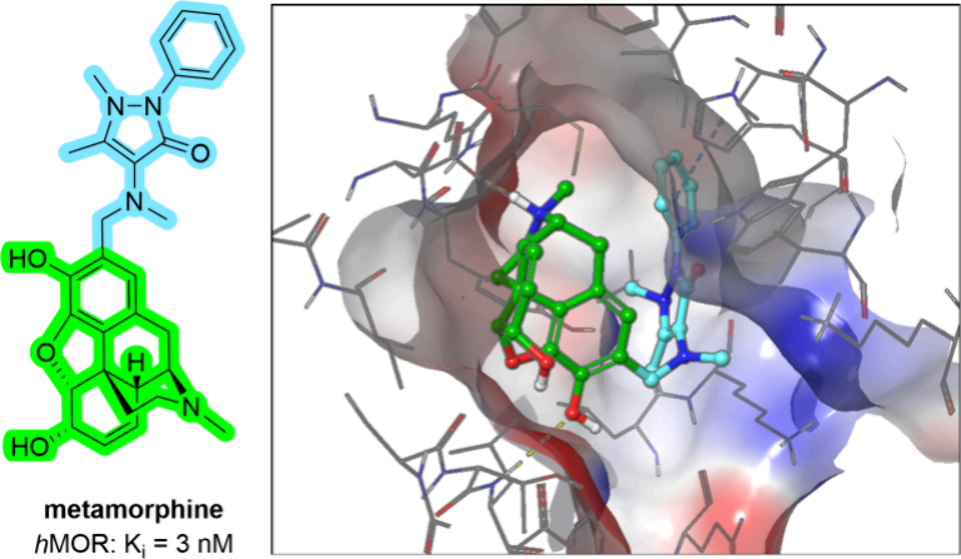

Opioids are among the most effective drugs in managing moderate to severe pain. Herein, we describe a morphine-metamizole adduct with a phenazone moiety added at position C2 of the morphinan backbone. This adduct, coined metamorphine, was first detected as a byproduct of the morphine-metamizole drug interaction in patient-controlled analgesia (PCA) pumps used for management of severe pain. In this study, metamorphine was successfully synthesized using the Mannich condensation. Qualitative high-performance liquid chromatography with ultraviolet detection (HPLC-UV) analysis was employed to confirm the identity of metamorphine, and the yield was then purified using preparative HPLC. Subsequent studies were undertaken to investigate the pharmacological properties of the newly synthesized compound. A radioligand-based binding assay demonstrated that metamorphine binds strongly to the μ-opioid receptor (*K_i_* = 3.0 nM). Functional assays showed that it activates both G-protein and β-arrestin pathways, with EC_50_ values of 0.169 and 3.06 μM, respectively. However, metamorphine did not exhibit significant activity on TNF, suggesting that it may lack analgesic, antipyretic, and anti-inflammatory effects associated with this pathway. Based on our findings, PCA pumps should be closely monitored, while therapeutic drug monitoring can be utilized to measure metamorphine serum concentration alongside with other opioids. Lead optimization of metamorphine could potentially result in new opioids with an expanded pharmacological spectrum and/or reduced side effects.

Pain management constitutes a critical challenge within healthcare settings. Its effective mitigation is essential for enhancing patient well-being and overall quality of life, while concurrently facilitating recovery and rehabilitation.^1−5^ Opioid analgesics have historically been regarded as the primary modality for pain relief, attributable to their potent effects on the central nervous system’s nociceptive pathways. The ability of these pharmacological agents to rapidly and substantially reduce pain has reinforced their status as a cornerstone in the management of both acute and chronic pain syndromes.^6−8^ However, the analgesic benefits of opioid medications are partially offset by significant adverse effects, including the potential for addiction, which presents a serious risk to patients and has contributed to widespread public health crises in various regions.^9−11^

Patient-controlled analgesia (PCA) pumps are commonly used to improve pain control across different patient populations, including managing postoperative pain, palliative care or patients suffering from acute or chronic pain conditions.^12−19^ Combining opioids (e.g., morphine) with other analgesics (e.g., metamizole) is believed to reduce the side effects associated with monotherapy PCA pumps and improve pain management. A major drawback of morphine-metamizole PCA pumps is the stability of the drug mixture. At 37 °C, the concentration of morphine significantly decreases, dropping to approximately 72% of its initial concentration in the PCA pump after 7 days.^20^ Moreover, metamizole sodium is a prodrug that hydrolyzes in water or methanol—slowly at neutral pH and more rapidly in acidic conditions—yielding its primary active metabolite, 4-methylaminoantipyrine (4-MAA).^21−23^ This unexpected decrease of both drugs is due to a drug interaction that leads to the *in situ* formation of a new substance, coined “metamorphine”, through the Mannich condensation. In this reaction, the phenolic ring of morphine undergoes electrophilic substitution at position C2, facilitated by an iminium ion formed from 4-MAA and formaldehyde, both sourced from metamizole, leading to the incorporation of a phenazone-derived aminomethyl moiety into the morphinan scaffold.

Although morphine-metamizole PCA pumps are widely used in managing pain in palliative care, it was not reported any loss of efficacy over time, due to the formation of metamorphine, thus warranting further investigations of its pharmacological effects.^24^ In this study, we present the synthesis and *in vitro* biological evaluation of metamorphine, serving an exploratory study for the addition of active moieties to the morphinan backbone of morphine. Since morphine is a potent and selective agonist of the human μ-opioid receptor (MOR),^25^ we therefore evaluated metamorphine in comparison to two other μ-opioid agonists (Figure 1), namely [Met^5^]enkephalin, [d-Ala,^2^N-Me-Phe,^4^Gly^5^-ol]enkephalin (DAMGO), and TRV-130 (oliceridine), in both *in vitro* MOR binding and activation assays. The discovery of metamorphine paves the way for a new class of opioids with a broader spectrum of activity. Polypharmacology, or multitargeting, is an approach in drug design that aims to develop compounds capable of targeting multiple receptors.^26,27^ This strategy has several advantages over conventional polypharmacy, including enhanced patient compliance by reducing the number of administered drugs, particularly in some complex diseases and multimorbid patients.^28^

**Figure 1:**
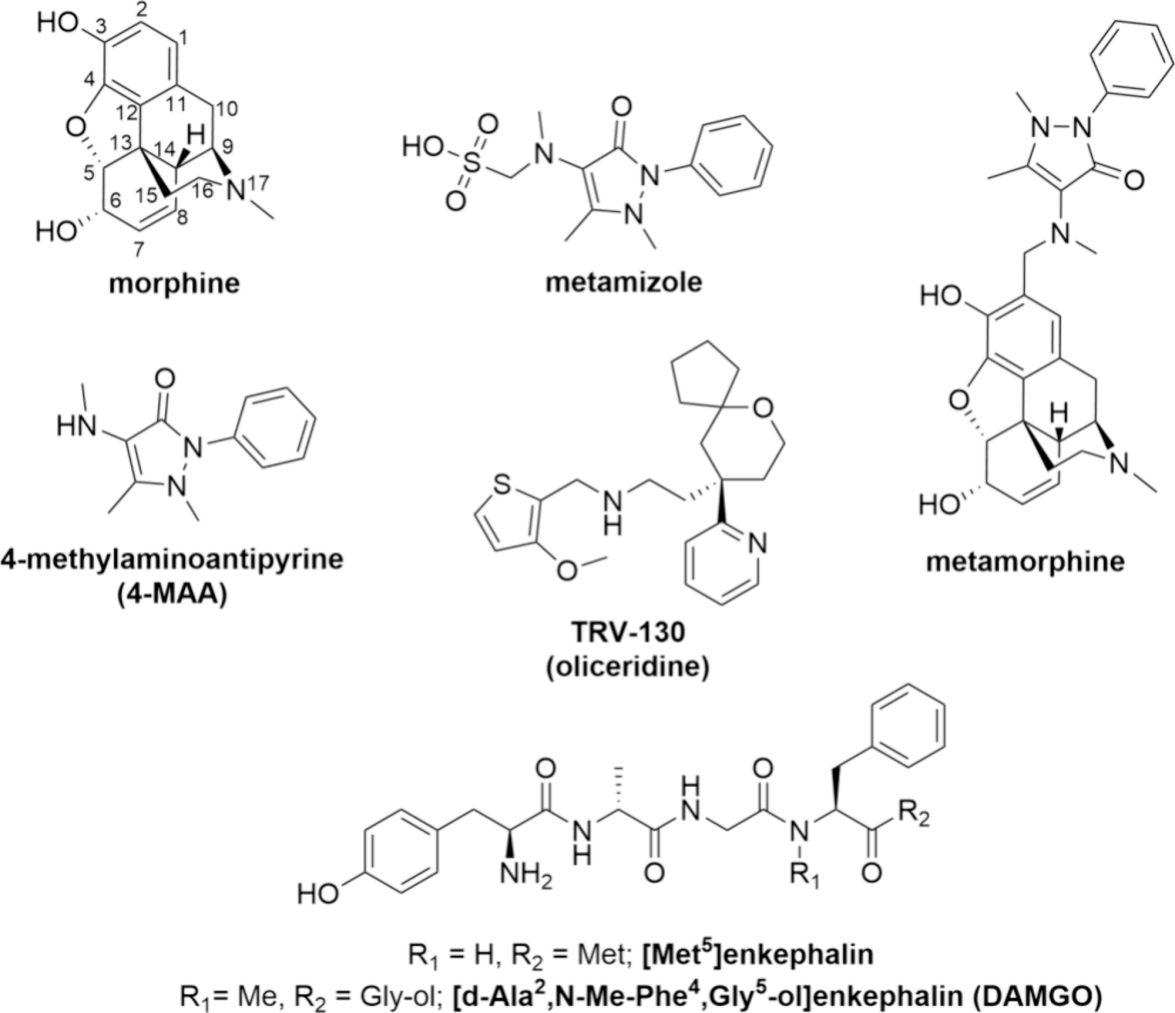
Chemical structures of compounds used in this study.

It is anticipated that metamizole retains the analgesic properties of morphine while also incorporating the analgesic and mild anti-inflammatory characteristics of 4-MAA. Such a dual-action profile could offer significant benefits in managing postoperative pain, a condition that poses substantial clinical challenges. Current clinical guidelines recommend combining opioids with nonsteroidal anti-inflammatory drugs (NSAIDs) for effective pain relief. Suboptimal postoperative pain management can lead to the development of chronic postoperative pain, negatively impacting patient recovery and quality of life.^29^ Thus, developing new opioids that address both pain and inflammation could significantly improve outcomes for patients undergoing surgical procedures.

## Results and Discussion

### Chemistry

The formation of metamorphine in patient-controlled analgesia (PCA) pumps follows the Mannich reaction, a multicomponent condensation reaction involving formaldehyde, an amine, and a compound with an active hydrogen atom. This reaction is used to create β-amino-carbonyl products with new carbon–carbon bonds.^30,31^

**Scheme 1:**
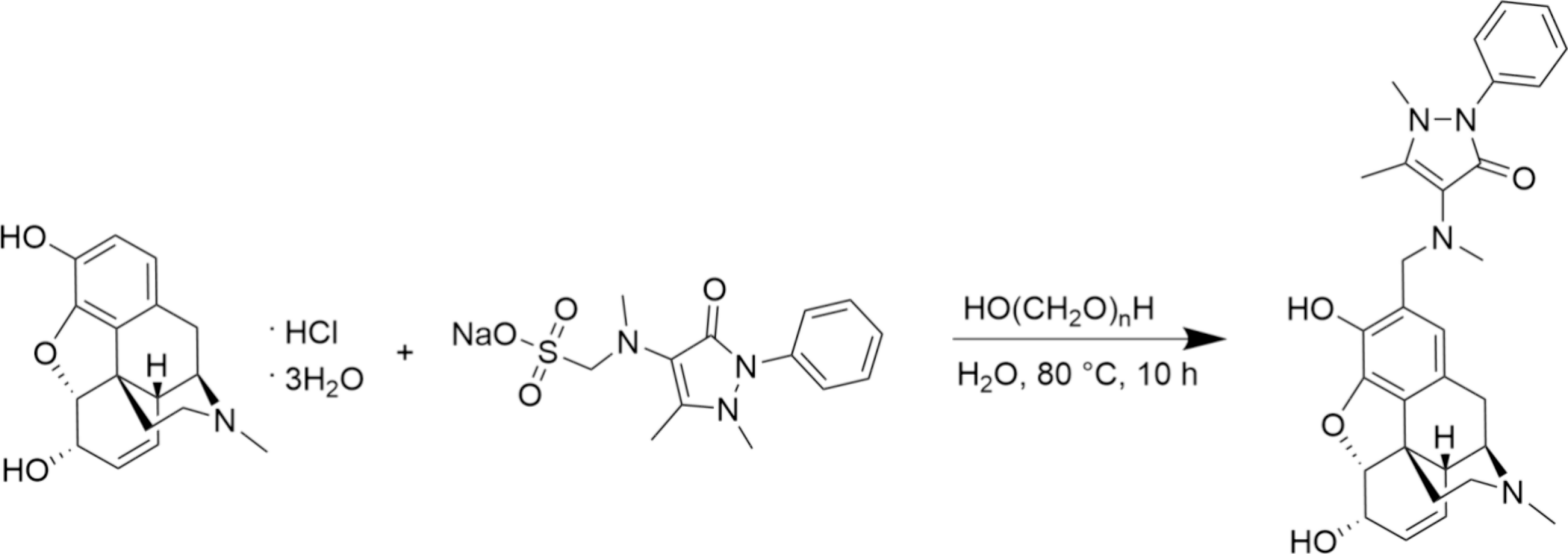
Synthesis of Metamorphine

In the present work, morphine hydrochloride trihydrate was mixed with metamizole sodium and paraformaldehyde in water, mimicking the components of the PCA pumps. The reaction conditions were modified to accelerate the formation of metamorphine by heating the reactants at 80 °C for 10 h (Scheme 1). During this reaction, position C2 on the phenolic ring of morphine acts as the nucleophilic site providing the activated carbon; 4-MMA serves as the secondary amine; and paraformaldehyde acts as the electrophilic carbon source, facilitating the formation of the aminomethyl bridge between the morphine and 4-MMA. We tested various reaction conditions to optimize the synthesis of metamorphine and found that using a 5:1 mass ratio of metamizole sodium to morphine hydrochloride trihydrate resulted in the fastest reaction time and complete conversion of morphine to metamorphine.

**Figure 2:**
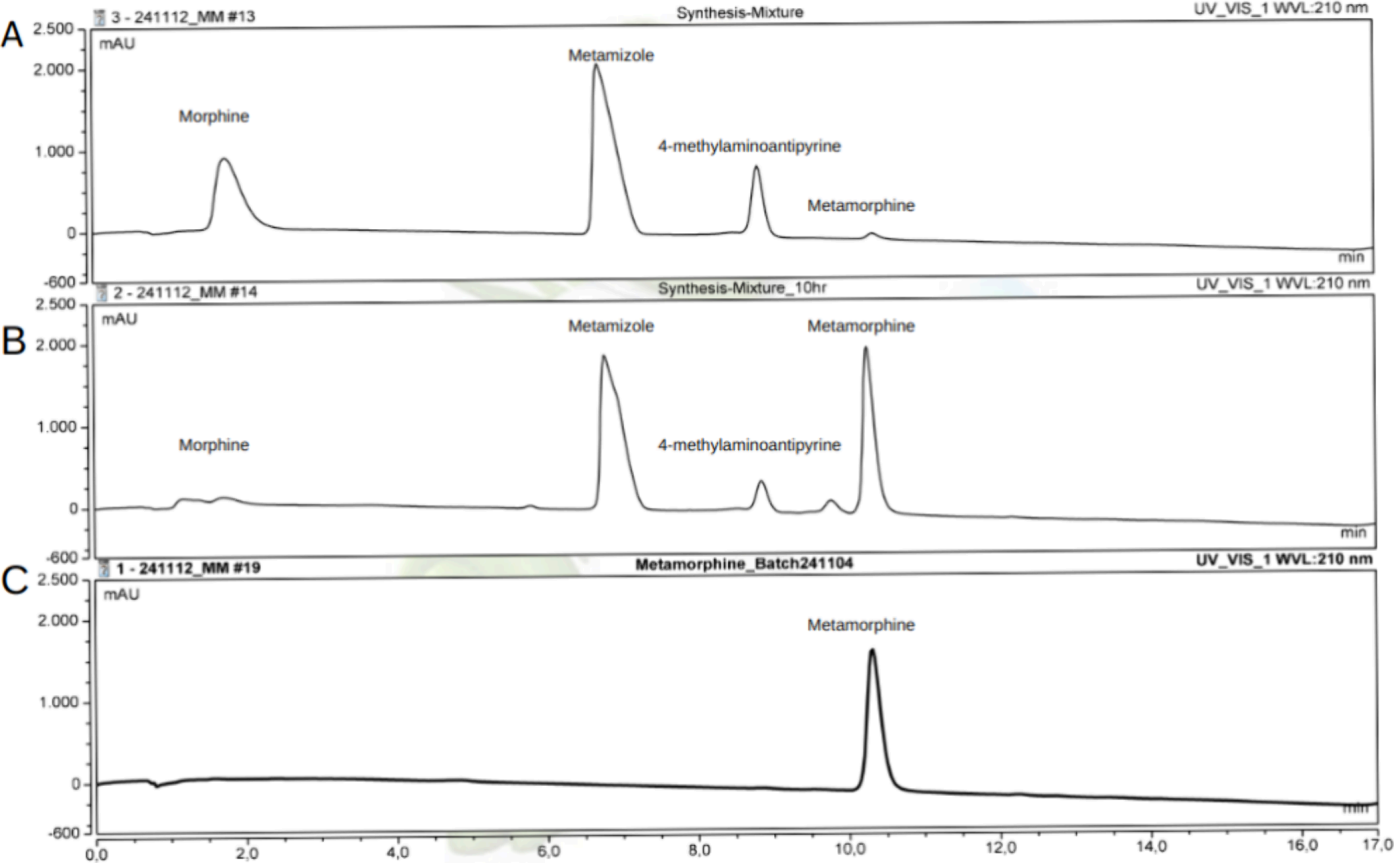
Analytical HPLC for metamorphine synthesis. (A) Chromatogram of the initial synthesis mixture, showing the reactants. (B) Chromatogram after 10 h of reaction at 80 °C, indicating the formation of metamorphine. (C) Chromatogram of the isolated metamorphine peak, successfully separated from the synthesis mixture in methanol.

The product was subsequently purified and isolated from the reactants using reverse-phase high-performance liquid chromatography (HPLC). An analytical HPLC-UV method was optimized to achieve efficient separation of the synthesis mixture components. The chromatographic conditions provided baseline separation of morphine, metamizole, 4-MAA, and metamorphine. The retention times for these compounds were 1.8, 6.8, 8.8, and 10.6 min, respectively (Figure 2A & B). Although the chosen reactant ratio was optimal for synthesis, it posed challenges for isolating metamorphine from the reaction mixture due to the excess concentration of metamizole, as shown in Figure 2B. To address this, a preparative HPLC method was developed based on the analytical method to effectively isolate metamorphine from the synthesis mixture. The collected fractions contained pure metamorphine, confirmed by reanalysis with the analytical HPLC method (Figure 2C). The preparative method allowed for the collection of metamorphine starting from retention time of 20.5 min and for 8 min.

The collected fraction was pulverized using nitrogen gas to evaporate methanol. The identity of the resulting powder as metamorphine was confirmed through nuclear magnetic resonance (NMR) spectroscopy and high-resolution mass spectrometry (HRMS). The spectral data of the synthesized metamorphine (Supplementary Figures S1–4) were compared to that of the morphine-metamizole adduct produced *in situ* in PCA pumps,^31^ revealing that both compounds are identical.

### Pharmacology

#### *In Vitro* Radioligand Competition Binding Assay

**Figure 3:**
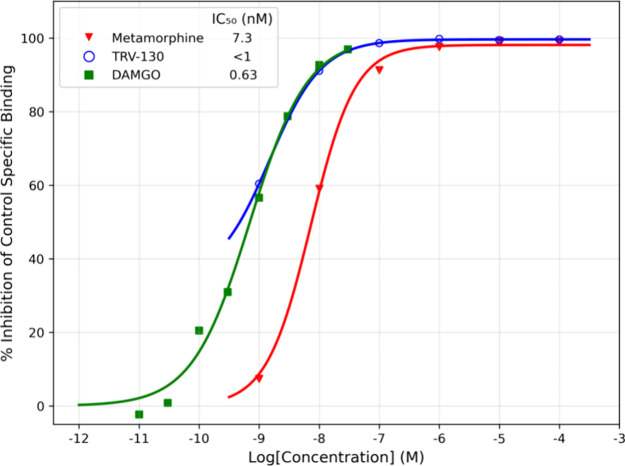
Competitive displacement binding curves for metamorphine and TRV-130 binding to MOR.

To determine whether metamorphine binds within the orthosteric pocket of MOR, radioligand competitive binding studies against [^3^H]DAMGO were performed. The results indicated that metamorphine binds strongly to the MOR with a half-maximal inhibitory concentration (IC_50_) of 7.3 nM corresponding to an inhibitory constant (K_i_) of 3.0 nM (Figure 3). Not surprisingly, metamorphine is about 8-fold less potent against MOR than morphine, which has a reported K_i_ = 0.37 nM.^32^ In comparison, TRV-130 (a G-protein biased MOR agonist) exhibited an IC_50_ < 1.0 nM (K_i_ not determined). Additionally, the reference compound DAMGO displayed an IC_50_ = 0.63 nM (K_i_ = 0.26 nM). These results indicate that metamorphine’s binding affinity is comparable to that of established MOR agonists, despite the phenazone moiety, which initially raised concerns about steric hindrance. The binding assays confirm that metamorphine fits well within the MOR’s binding pocket while retaining its pharmacological efficacy.

#### *In Vitro* Functional Assays

**Figure 4:**
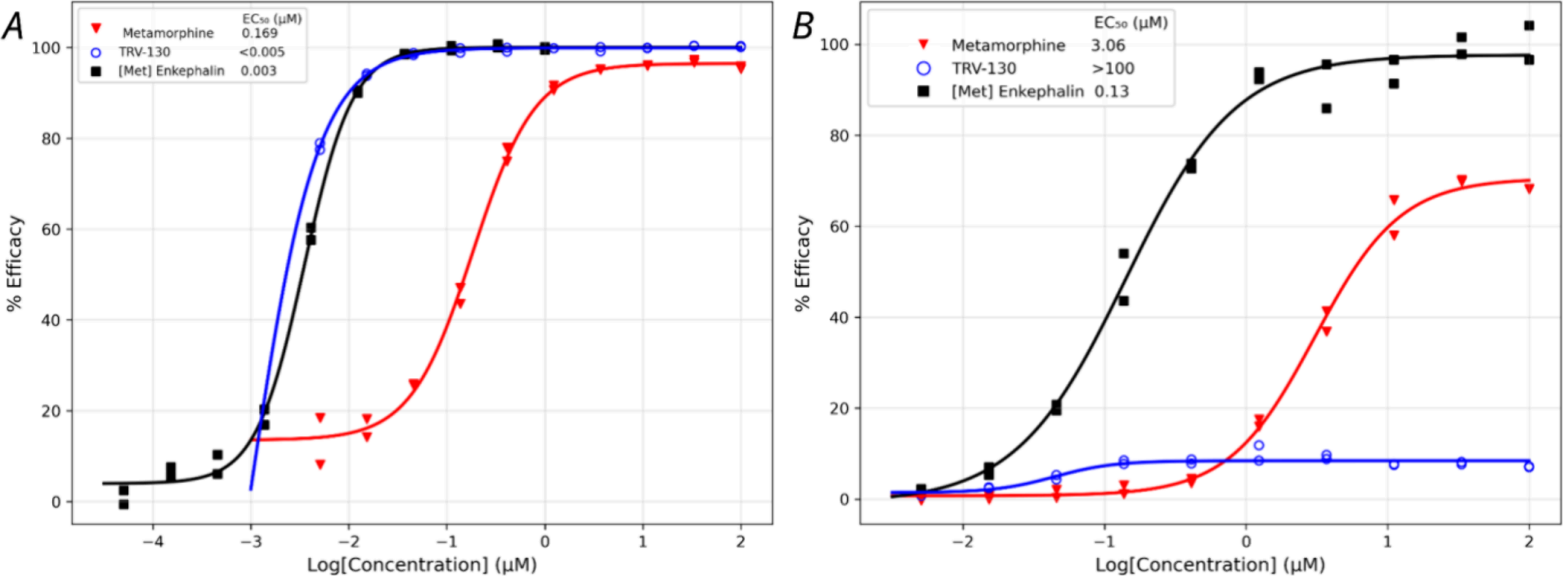
Concentration–response curves for three MOR agonists on signaling pathway activation. (A) G-protein pathway and (B) β-arrestin pathway.

Following the confirmation of metamorphine’s binding to the MOR, we conducted a cyclic adenosine 3′,5′-monophosphate (cAMP) accumulation assay and an enzyme fragment complementation (EFC) assay to evaluate its MOR activation and delineate the effects on G-protein and β-arrestin pathways, respectively. The results indicated that metamorphine activates both G-protein and β-arrestin pathways, with a half-maximal effective concentration (EC_50_) of 0.169 μM (*E*_max_ = 97%) for G-protein activation and EC_50_ = 3.06 μM (*E*_max_ = 70%) for β-arrestin recruitment (Figure 4). Morphine has been reported to activate both pathways with EC_50_ = 7.64 nM (*E*_max_ = 103.5%) for G-protein activation and EC_50_ = 0.383 μM (*E*_max_ = 33%) for β-arrestin recruitment.^33^ A similar trend (8-fold difference) is observed for β-arrestin recruitment of metamorphin compared to morphine, however with a 22-fold difference for the G-protein activation. Furthermore, [Met^5^]enkephalin, the endogenous ligand and a full agonist of MOR, displayed EC_50_ = 3 nM (*E*_max_ = 100%) for G-protein activation and EC_50_ = 0.13 μM (*E*_max_ = 100%) for β-arrestin recruitment. In these signaling assays, the *E*_max_ value of an agonist reflects its intrinsic efficacy.^34^ Thus, our findings indicate that, like morphine and [Met^5^]enkephalin, metamorphine is not a biased agonist, particularly in contrast to the G-protein biased agonist TRV-130 which showed EC_50_ < 5 nM (*E*_max_ = 100%) for G-protein activation and EC_50_ > 100 μM (*E*_max_ = 7.9%) for β-arrestin recruitment.

#### *In Vitro* Inflammatory Cytokines Assay

**Figure 5:**
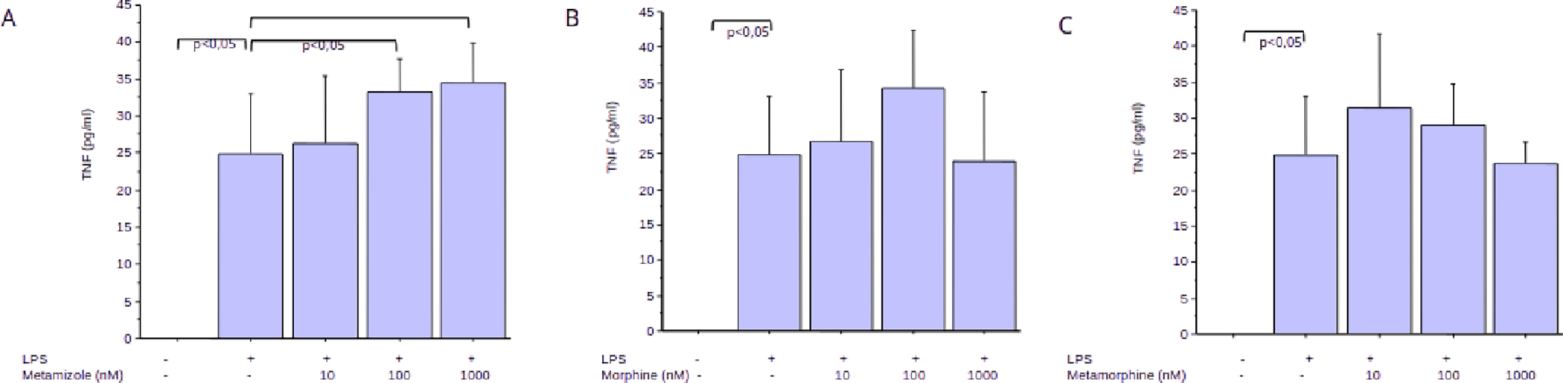
Interaction of the three analgesics with LPS stimulation. (A) Metamizole induces a dose-dependent increase of the LPS-induced (0.1 μg/mL) TNF release. This effect reaches significance in the higher doses (*p* < 0.05). In contrast, no significant effect was seen using (B) morphine or (C) metamorphine.

In order to study possible interactions with pro-inflammatory stimuli, we treated either nonactivated or lipopolysaccharide(LPS)-activated (0.1 μg/mL) THP-1 cells with metamizole sodium, morphine, and metamorphine. As expected, in nonactivated cells, we did not see any induction of pro-inflammatory cytokines, including the tumor necrosis factor (TNF), interleukin-1 beta (IL-1beta), or interleukin 6 (IL-6) by metamizole, morphine or metamorphine. Conversely, metamizole induced a dose-dependent and significant upregulation of LPS-induced TNF release (Figure 5A). This effect is primarily attributed to the inhibition of prostaglandin E_2_ (PGE_2_)-induced hyperalgesia.^21−23,35,36^ Similar effects could not be observed using morphine or metamorphine (Figure 5B & C). Moreover, no effects could be observed by all three compounds on either IL-1beta or IL-6 release (Supplementary Figures S5 & S6). This suggests that its relatively large structure of metamorphine may hinder interaction with cyclooxygenase enzymes. Another hypothesis suggests the involvement of the cannabinoid CB_1_ receptor in metamizole-induced analgesia.^36^ Therefore, testing metamorphine against cannabinoid receptors could be envisioned.

### Molecular Modeling

**Figure 6:**
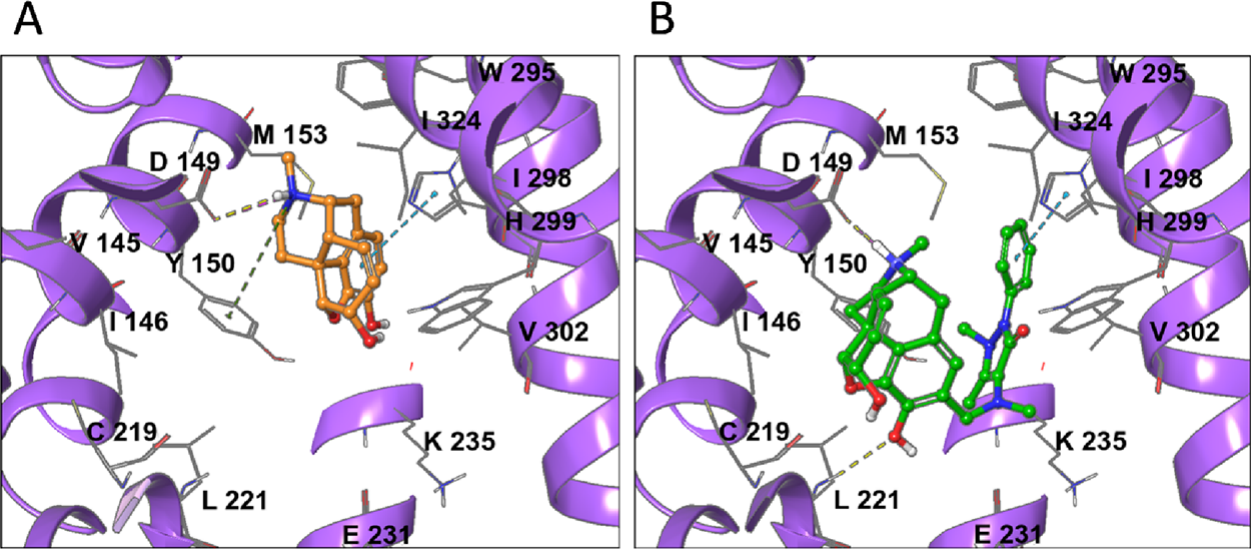
Binding mode of morphinans in the MOR orthosteric site. (A) Cryo-EM model of morphine (PDB ID: 8EF6). (B) Docking model of metamorphine.

Starting off with the first cryo-electron microscopy (cryo-EM) structure of MOR complexed with morphine (Figure 6A; PDB ID: 8EF6), recently published by Zhuang et al.,^37^ we employed molecular docking to predict the potential binding mode of metamorphine (Figure 6B). Although both compounds share the same morphinan scaffold, they exhibit different binding modes within the MOR orthosteric site. In the cryo-EM structure, morphine forms a cation-π interaction with Y150, engages in π–π stacking with H299, and establishes an H-bond and salt bridge with D149. The interaction with D149 is essential for the binding and activation of MOR signaling pathways by both natural and synthetic opioids. In contrast, metamorphine forms an H-bond and a salt bridge with D149, while its π–π stacking interaction with H299 occurs through the phenazone moiety, rather than the morphinan phenolic ring as seen with morphine. Moreover, it establishes an H-bond with L221. The predicted bing mode of metamorphine indicates that position C2 is not the optimal attachment point for large moieties, such as phenazone, as it causes the morphinan scaffold to flip. This could explain the reduced binding affinity and activation of MOR by metamorphine.

## Conclusion

Based on our findings, PCA pumps should be closely monitored, while therapeutic drug monitoring can be utilized to measure metamorphine serum concentration alongside with other opioids. This is an essential step to prevent opioid-related side effects and overdosing, given that metamorphine effectively activates MOR. Demonstrating that the addition of a new moiety to position C2 on the morphinan backbone does not compromise its activity at the MOR could be pivotal in designing new opioids with reduced side effects. However, the phenazone moiety within metamorphine did not exhibit significant effects on TNF release, suggesting that metamorphine may not replicate the analgesic and antipyretic effects of metamizole. Future studies should explore additional molecular modifications and target other opioid receptors to optimize the therapeutic profile of this new class of compounds. Continued research in this area could lead to the development of more effective opioids with enhanced polypharmacological activity and reduced side effects.

## Materials and Methods

### Chemistry

All solvents used were of HPLC grade. Methanol was procured from VWR International, while ultrapurified sterile water was sourced from B. Braun Melsungen AG. Formic acid and ammonia, utilized in buffer preparation, were obtained from Carl Roth GmbH + Co. KG. Morphine hydrochloride trihydrate was acquired from Th. Geyer GmbH & Co. KG, and metamizole sodium was supplied by Ratiopharm GmbH for the synthesis and by Caesar & Loretz GmbH for the *in vitro* biological assay. TRV-130 was provided by TargetMol Chemicals Inc. Analytical and preparative chromatography were performed using a Dionex UltiMate 3000 system (Thermo Fisher Scientific Inc.), equipped with a diode array detector (DAD) and an automated fraction collector. NMR spectra were measured on a Bruker Avance II 400 MHz spectrometer and the spectra were analyzed with MestReNova 14.2.3. The chemical shift δ was given in part per million (ppm) and the coupling constant J in Hertz (Hz). ^1^H NMR and ^13^C NMR spectra were referenced to an internal solvent signal standard as described in literature. High resolution mass spectra (HRMS) were recorded using a Thermo LCQ Advantage.

### Synthesis of Metamorphine

Morphine hydrochloride trihydrate (44 mM) and metamizole sodium (267 mM) were mixed in water. Paraformaldehyde (61 mM) was then added to the reaction mixture. The components were heated at 80 °C for 10 h and the reaction progress was monitored by HPLC-UV. Upon completion of the reaction, the mixture was cooled, and the product was purified by preparative HPLC. Yellowish white powder (110 mg, Yield: 40%). ^1^H NMR (400 MHz, DMSO-*d*_*6*_) δ 8.34 (s, 1H), 7.54–7.42 (m, 2H), 7.39–7.24 (m, 4H), 6.26 (s, 1H), 5.54 (dddd, J = 9.7, 3.2, 2.0, 1.3 Hz, 1H), 5.22 (ddd, J = 9.8, 3.2, 2.3 Hz, 1H), 4.69 (dd, J = 6.1, 1.3 Hz, 1H), 4.09 (ddt, J = 5.9, 3.2, 2.3 Hz, 1H), 3.96 (d, J = 12.9 Hz, 1H), 3.87 (d, J = 12.8 Hz, 1H), 3.28 (dd, J = 6.2, 3.2 Hz, 1H), 2.91–2.81 (m, 1H), 2.64 (s, 3H), 2.55 (d, J = 2.8 Hz, 1H), 2.56–2.43 (m, 1H), 2.32 (s, 3H), 2.32–2.25 (m, 1H), 2.28–2.17 (m, 1H), 2.21–2.13 (m, 1H), 2.00 (s, 3H), 2.07–1.93 (m, 1H), 1.68–1.60 (m, 1H). ^13^C NMR (101 MHz, DMSO-*d*_*6*_) δ 164.70, 162.92, 151.12, 137.36, 134.97, 133.20, 129.78, 128.74, 127.95, 125.65, 125.08, 124.06, 122.79, 119.98, 91.37, 66.01, 57.82, 55.05, 45.70, 42.42, 42.34, 40.23, 40.09, 39.75, 39.54, 36.07, 34.96, 19.91, 9.95. HRMS (ESI): calculated for C_30_H_34_N_4_O_4_ [M + H]+, 515.2580; found, 515.2661. HPLC purity: 90%.

### Analytical HPLC

HPLC was utilized to analyze the synthesis mixture and confirm the identity of purified metamorphine. Reversed-phase chromatography (RPC) was employed to achieve peak separation within the synthesis mixture. The total run time of the method was 17 min. The mobile phase consisted of methanol and ammonium formate buffer (pH 5). Flow rate was set at 0.5 mL/min throughout the analysis. Reprosil Pur Basic-C18 column (100 × 2 mm, 3 μm) was used with LiChroCART 4–4 Purospher RP-18 (5 μm) as a guard column. Their temperature was maintained at 40 °C throughout the analysis. 0.1 μL of the synthesis mixture was injected. The gradient program started with 5% methanol, increasing linearly to 40% over 15 min, followed by a 2 min wash phase.

### Preparative HPLC

The preparative HPLC method was designed to isolate metamorphine from the synthesis mixture. Conditions mirrored those of the analytical method, with adjustments for the Reprosil Pur Basic-C18 column (150 × 10 mm, 5 μm) and an increased flow rate of 2.5 mL/min. The gradient began with 25% methanol, increasing to 30% over 20 min, and subsequently to 100% over the next 10 min, during which metamorphine was collected. The gradient then returned to 25% methanol for a 10 min wash phase, concluding the run at 40 min. For each analysis and synthesis, a fresh 10 mM ammonium formate buffer was prepared. The pH of the buffer was adjusted to 5 using a calibrated pH meter.

### Pharmacology

#### *In Vitro* Radioligand Competition Binding Assay

The binding assay was performed at Eurofins utilizing MOR expressed in human embryonic kidney 293 (HEK-293) cells, as previously described.^38^ Briefly, the binding affinities of metamorphine and TRV-130 were determined by assessing the percent inhibition of [^3^H]DAMGO, a radiolabeled ligand. This assay was run in duplicate with DAMGO used as a reference compound. The K_i_ values were determined from the measured IC_50_ values using the Cheng-Prusoff equation,^39^ K_i_ = IC_50_/[1 + ([L]/K_d_)], where [L] and K_d_ are the concentration and binding affinity of the radiolabeled ligand in the assay, respectively.

#### *In Vitro* Functional Assays

Functional assays (HitHunter cAMP and PathHunter β-arrestin assays) were conducted at Eurofins to evaluate the activation of primary signaling pathways, specifically G-protein and β-arrestin, by metamorphine and TRV-130. These assays were run in duplicate with [Met^5^]-enkephalin was used as a reference compound. These assays aimed to determine the efficacy and pathway specificity of metamorphine in comparison to the known G-protein biased agonist, TRV-130.

##### HitHunter cAMP Assay

The G-protein pathway activation was performed as previously described.^40^ Briefly, the G-protein pathway activation was measured using HitHunter cAMP assays, which quantify cAMP, a secondary messenger involved in G-protein-coupled receptor (GPCR) signaling. This assay employed Enzyme Fragment Complementation (EFC) technology with β-galactosidase (β-Gal) as the reporter enzyme. Upon activation, β-Gal produces a chemiluminescent signal that is detected using a microplate reader, providing a quantitative measure of G-protein pathway activity.

##### PathHunter β-Arrestin Assay

The β-Arrestin recruitment assay was performed as previously described.^41^ Briefly, the PathHunter β-Arrestin assay was used to monitor GPCR activation through β-arrestin recruitment. This assay also utilized EFC technology, with β-Gal as the reporter enzyme. In this system, the Enzyme Acceptor (EA) fragment was fused to β-arrestin, and the ProLink (PK) fragment was fused to the GPCR of interest. Upon GPCR activation, EA and PK complementation restored β-Gal activity, generating a chemiluminescent signal that was measured using PathHunter Detection Reagents.

#### Inflammatory Cytokine Assay

This assay evaluated the impact of various compounds on cytokine production in THP1 cells stimulated with LPS, as previously described with some modifications.^35^ Following treatment, key cytokines were measured to enhance our understanding of the modulatory effects of these compounds on inflammation.

#### Stimulation of THP1 Cells

The human monocytic cell line THP1 (Signaling Factory Cell Line Bank, University of Freiburg) was cultured in RPMI 1640 (Gibco, ThermoFisher, Darmstadt, Germany). For the test, the cells were harvested, counted and adjusted to one million cells per milliliter in RPMI and either unstimulated or LPS stimulated and treated with morphine, metamizole, or metamorphine in three different concentrations as indicated in the results part. These assays were run in duplicate. The cells were cultured for 24 h at 37 °C and 5% CO_2_. After the culture period, the cell free supernatants were harvested and stored at −80 °C until cytokine measurement.

#### Cytokine Measurement

The cytokines IL-1beta, IL-6 and TNF were measured using an ELISA Development Kit (R&D, biotechne, Wiesbaden, Germany) according to the recommendations of the manufacturer.

### Statistics

Data are presented as mean ± standard deviation. Significance was calculated using Wilcoxon Signed Rank test,^42^ and accepted when *p* < 0.05. All calculations were performed using StatView 5.0.1 (SAS Institute Inc., Cary, NC, USA).

### Molecular Modeling

Modeling studies were carried out using the Schrödinger Small-Molecule Drug Discovery Suite 2024–4 (Schrödinger LLC, NY, USA). The X-ray crystal structure of MOR complexed with morphine (PDB ID: 8EF6) was retrieved from the RCSB PDB^43^ and prepared with Protein PrepWizard.^44^ This preparation included adding missing hydrogen atoms, adjusting ionization states of polar amino acids, modifying ligand bond orders and charges, optimizing hydrogen bonds, and energy-minimizing the complex with the OPLS4 force field.^45^ Metamorphine’s 3D Lewis structure was generated using LigPrep (Schrödinger LLC, NY, USA), involving ionization and tautomeric state generation at pH 7.4 and geometric optimization with OPLS4.^45^ Molecular docking was conducted with Glide^46^ utilizing the SP scoring function, with morphine’s geometric center as the grid centroid.

## Supporting Information Available

1H NMR, ^13^C NMR, ^1^H–^13^C HSQC NMR, HRMS spectra of metamorphine (PDF)
