# Supplementary material for: Synthesis and Biological Evaluation of Metamorphine: A Morphine–Metamizole Adduct from Patient-Controlled Analgesia Pumps
Source: ACS Pharmacol Transl Sci. 2025 Mar 3;8(3):718–25. doi: 10.1021/acsptsci.4c00546 (PMC11915030; doi:10.1021/acsptsci.4c00546)
Supplement: Supplementary file 1 — pt4c00546_si_001.pdf [file pt4c00546_si_001.pdf]

SUPPORTING INFORMATION

Synthesis and Biological Evaluation of Metamorphine: A Morphine-Metamizole Adduct from Patient-Controlled Analgesia Pumps

Aly Abotaleb[1,2], Aurélien F. A. Moumbock[2], Rainer Trittler[1], Gernot Zissel[3], Stefan Günther[2]*, Martin J. Hug[1]*

[1]Department of Pharmacy, University Medical Center and Faculty of Medicine, University of Freiburg, D-79106 Freiburg, Germany

[2]Institute of Pharmaceutical Sciences, Faculty of Chemistry and Pharmacy, University of Freiburg, D-79104 Freiburg, Germany

[3]Department of Pneumology, University Medical Center and Faculty of Medicine, University of Freiburg, D-79106 Freiburg, Germany

***Corresponding authors**

Aly Abotaleb; aly.abotaleb@uniklinik-freiburg.de

Stefan Günther; stefan.guenther@pharmazie.uni-freiburg.de

Table of contents

Figure S1. 1H NMR spectrum of metamorphine.....S1

Figure S2. 13C NMR spectrum of metamorphine.....S2

Figure S3. 1H–13C HSQC NMR spectrum of metamorphine..... S3

Figure S4. HRMS spectrum of metamorphine..... S4

Figure S5. IL-1beta showed no significant release after stimulation of THP-1 cells with LPS and no significant interaction of (A) morphine, (B) metamizole, and (C) metamorphine with the THP-1 cells..... S5

Figure S6. IL-6 showed no significant release after stimulation of THP-1 cells with LPS and no significant interaction of (A) morphine, (B) metamizole, and (C) metamorphine with the THP-1 cells.....S6

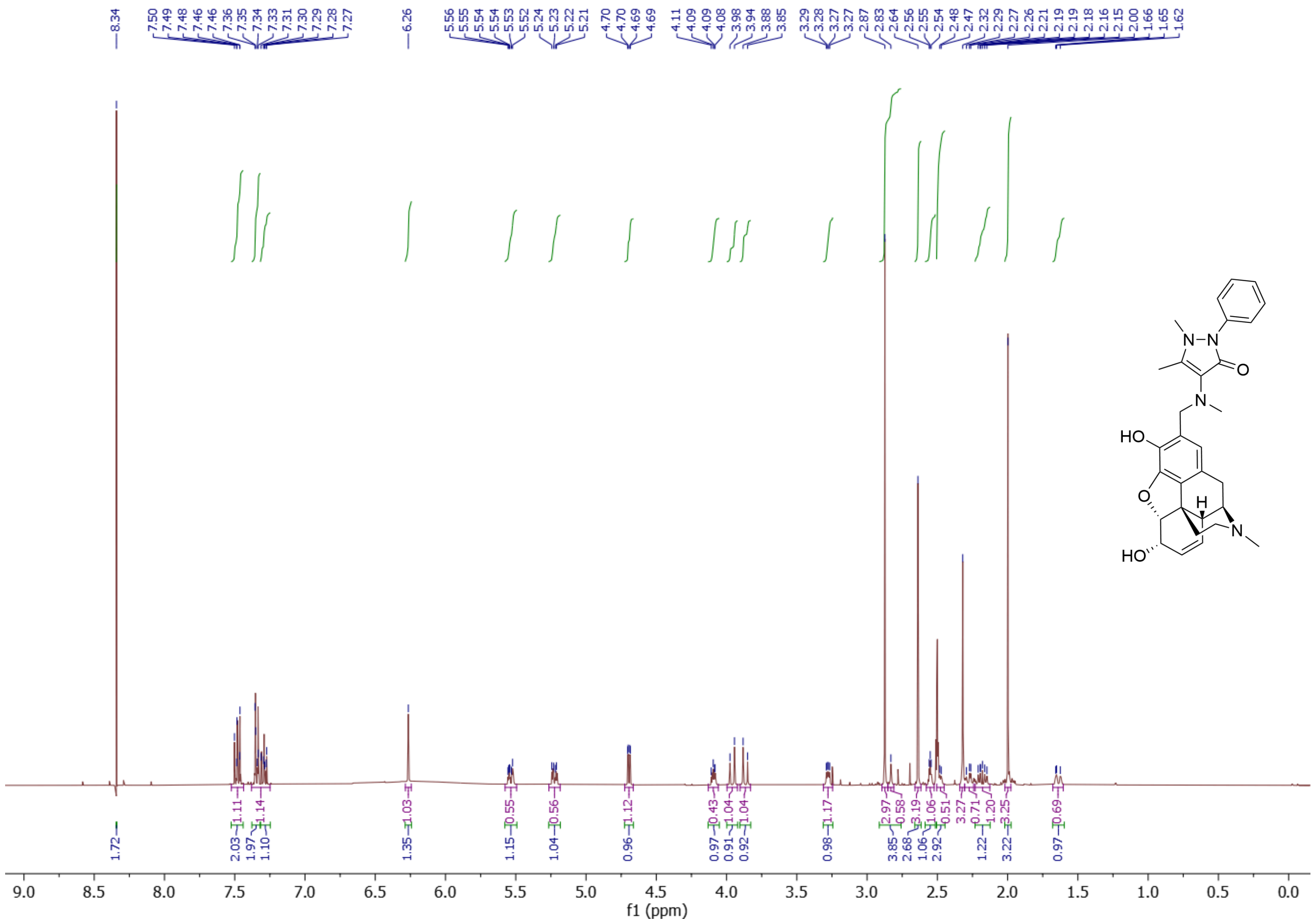

Figure S1. ^{1}H NMR spectrum of metamorphine

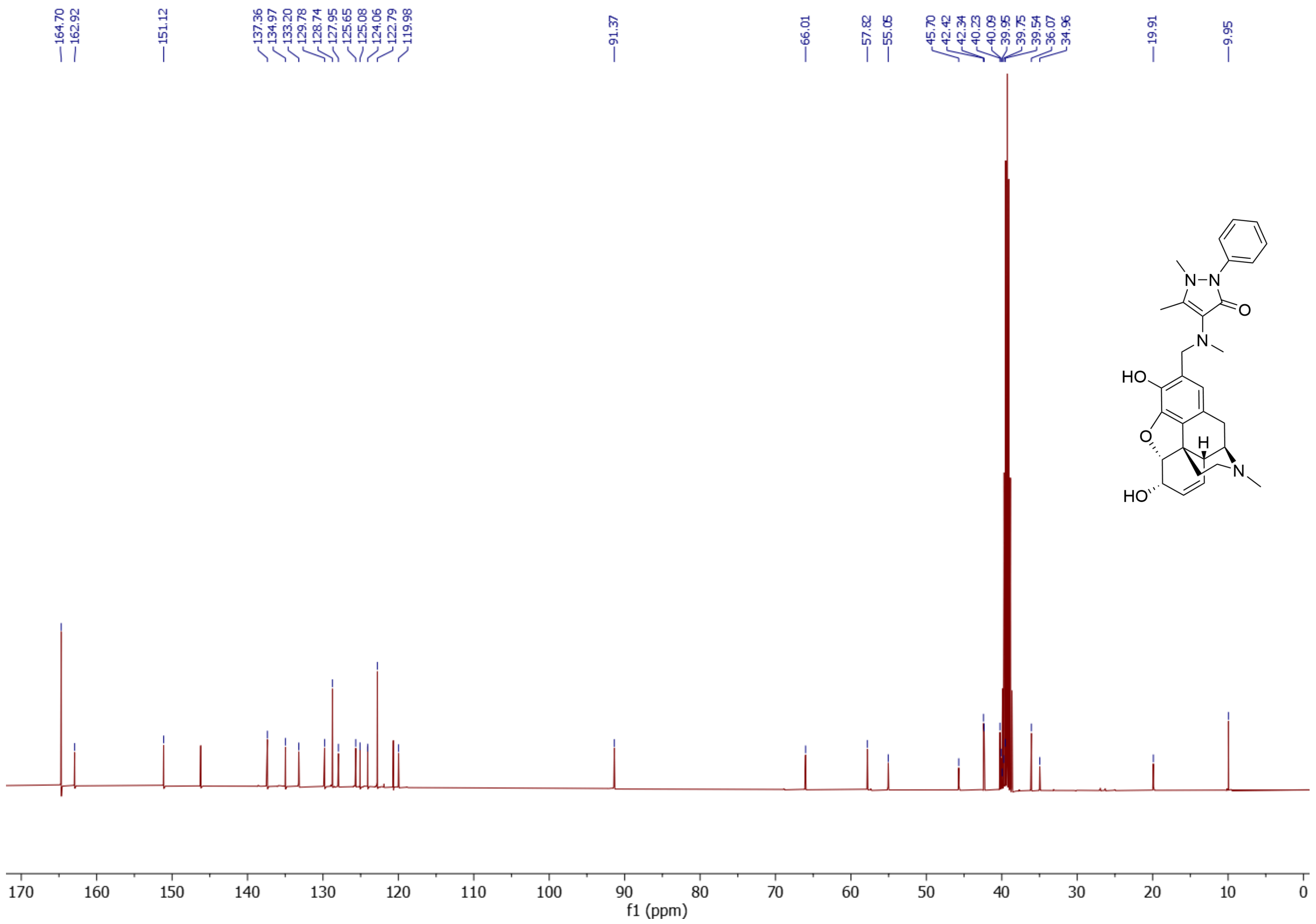

Figure S2. ^{13}C NMR spectrum of metamorphine

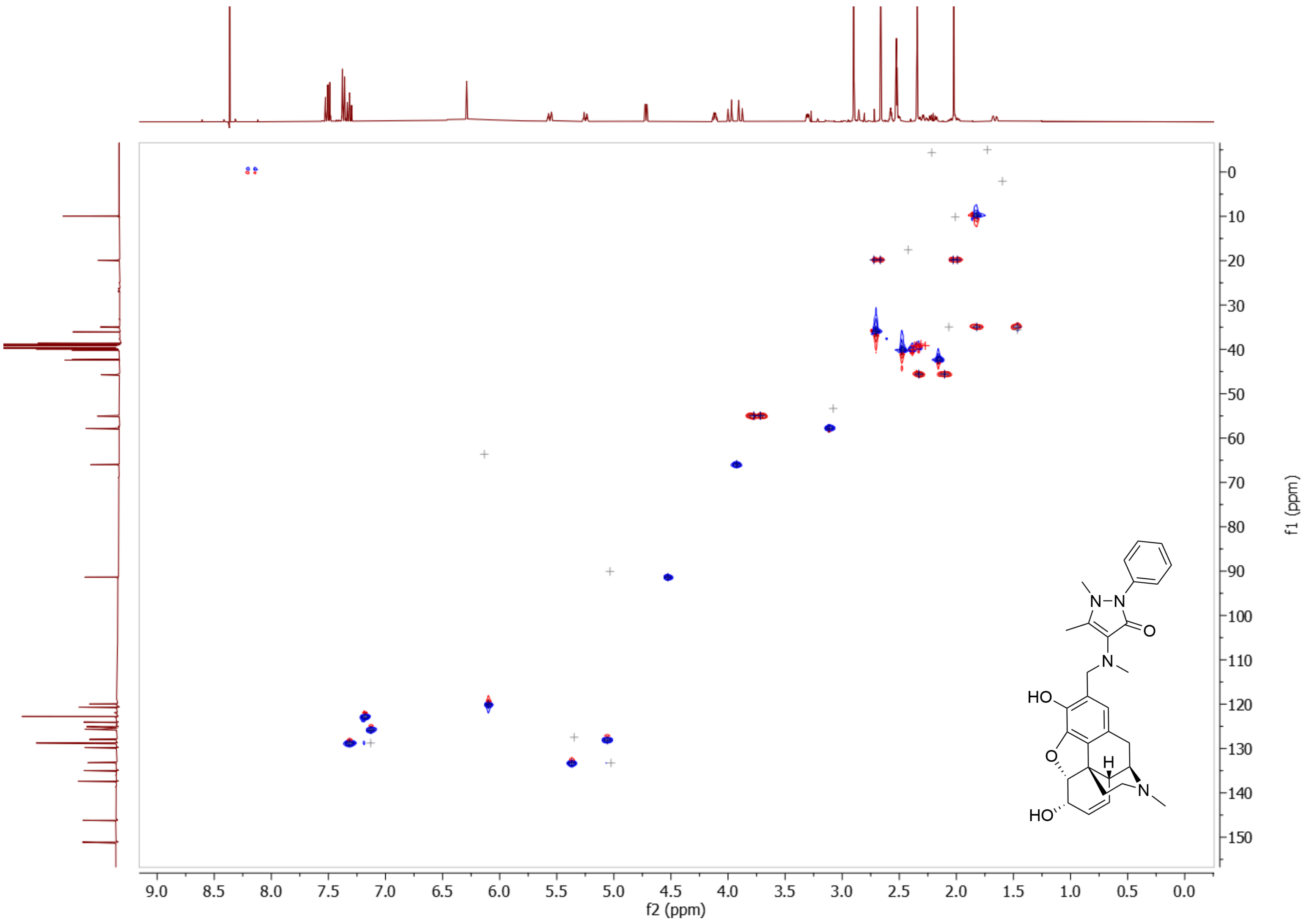

Figure S3. ^{1}H–^{13}C HSQC NMR spectrum of metamorphine

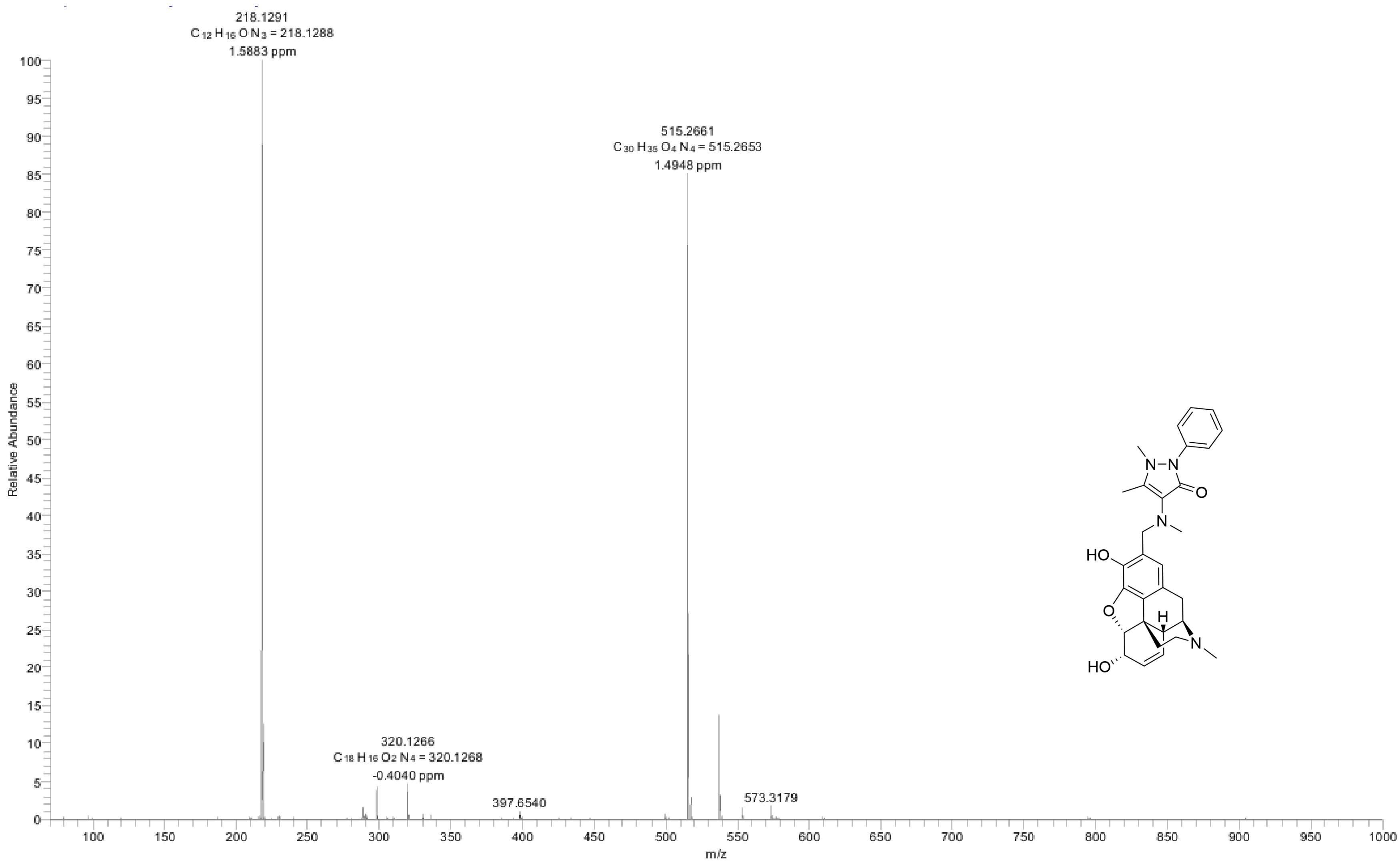

Figure S4. HRMS spectrum of metamorphine

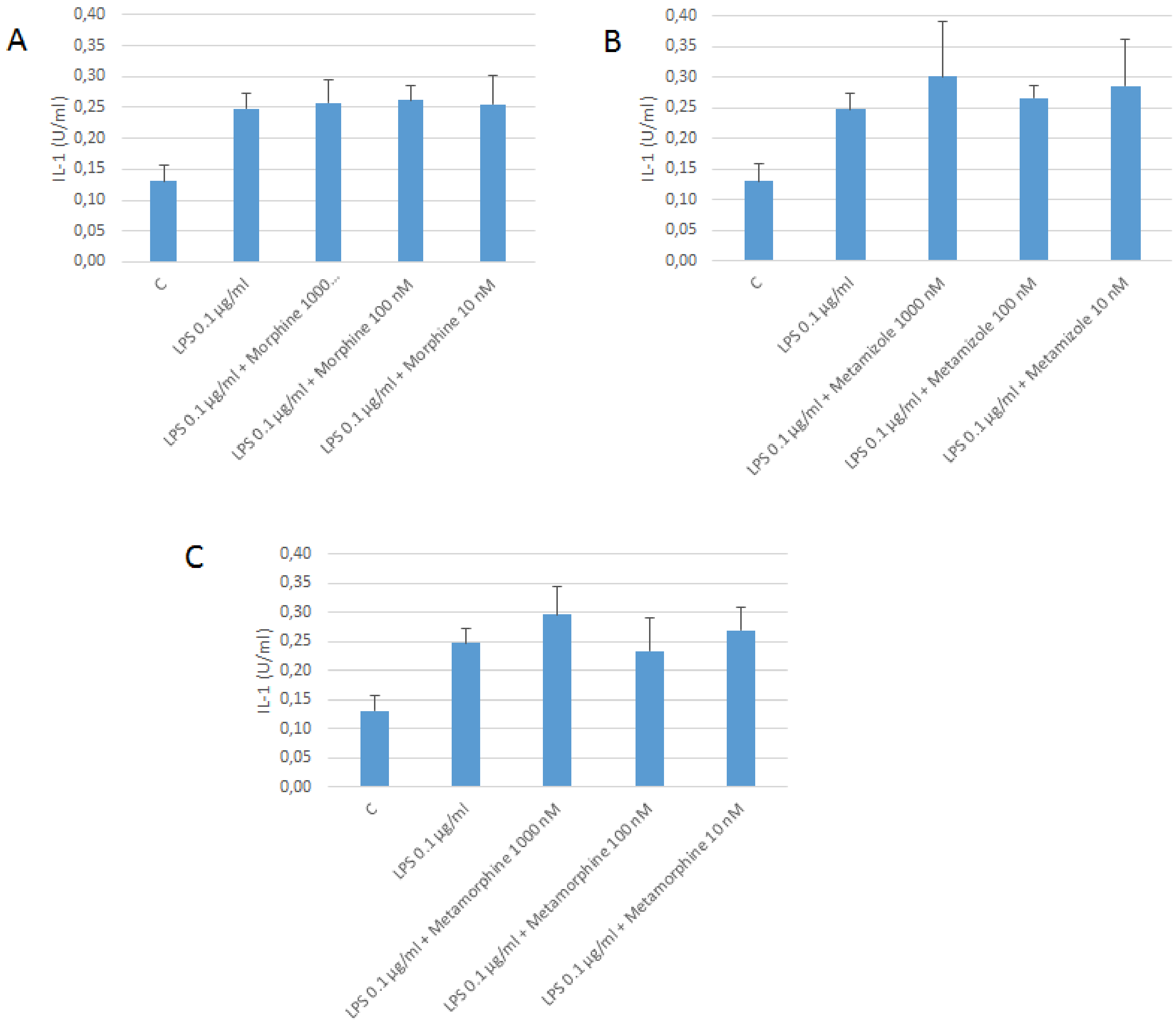

Figure S5. IL-1beta showed no significant release after stimulation of THP-1 cells with LPS and no significant interaction of (A) morphine, (B) metamizole, and (C) metamorphine with the THP-1 cells.

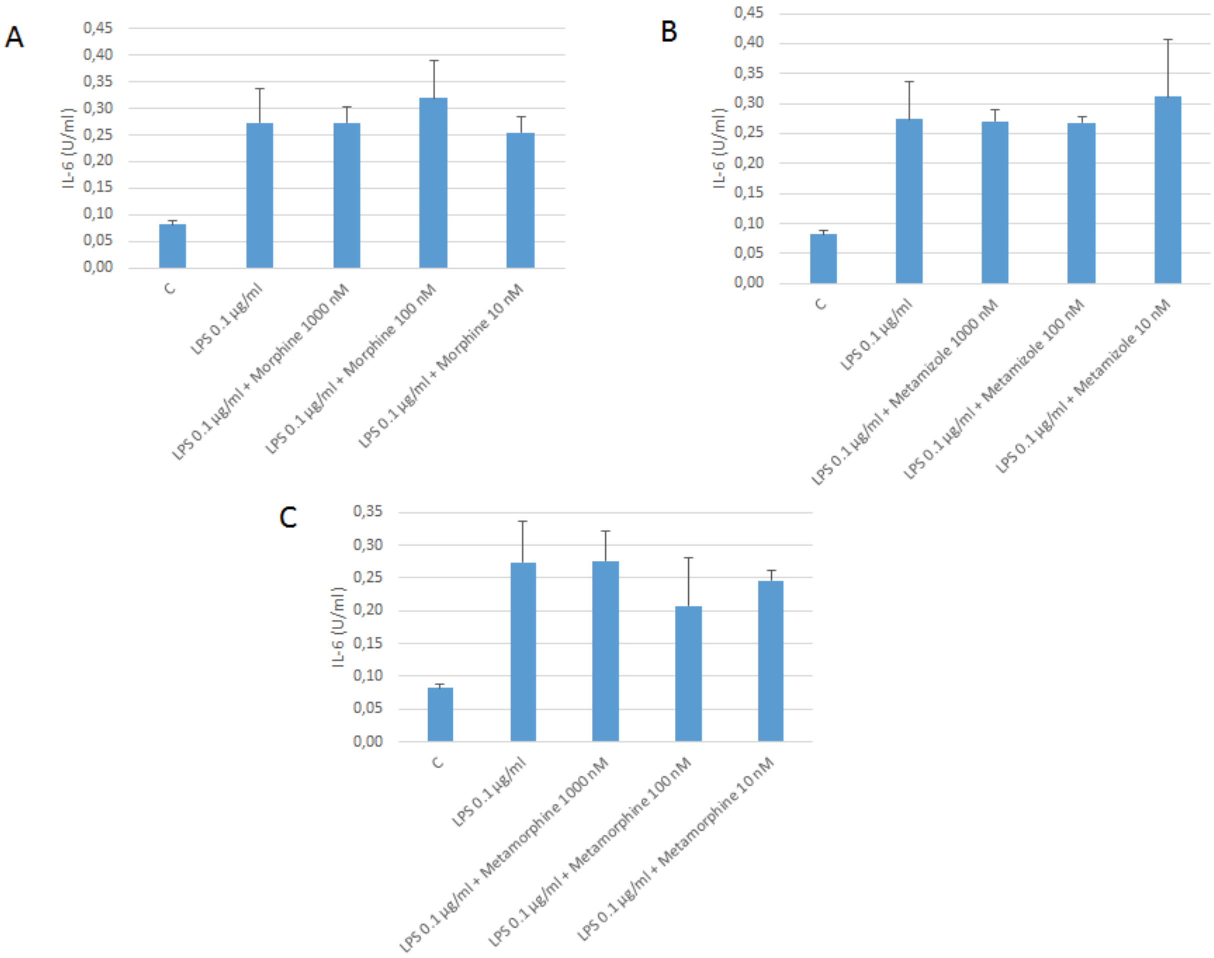

Figure S6. IL-6 showed no significant release after stimulation of THP-1 cells with LPS and no significant interaction of (A) morphine, (B) metamizole, and (C) metamorphine with the THP-1 cells.
